# The complete chloroplast genome of *Gaultheria fragrantissima* Wall. (Ericaceae) from Yunnan, China, an aromatic medicinal plant in the wintergreens

**DOI:** 10.1080/23802359.2021.1923425

**Published:** 2021-05-27

**Authors:** Xu Yan-Ling, Xin-Yu Du, Li Yi-Rong, Lu Lu

**Affiliations:** a School of Pharmaceutical Sciences and Yunnan Key Laboratory of Pharmacology for Natural Products, Kunming Medical University, Kunming, China; b Plant Germplasm and Genomics Center, Germplasm Bank of Wild Species, Kunming Institute of Botany, Chinese Academy of Sciences, Kunming, China

**Keywords:** Ericaceae, *Gaultheria fragrantissima*, methyl salicylate, phylogeny

## Abstract

Gaultheria fragrantissima (Ericaceae) is an aromatic medicinal plant with high concentrations of the secondarymetabolite methyl salicylate (oil of wintergreen). In this study, the complete chloroplast genomeof G. fragrantissima was sequenced. The complete plastome is 176,196 bp in length, and the GCcontent is 36.6%. The plastome comprises 110 unique genes (76 protein-coding, 30 tRNA and 4 rRNA).Phylogenetic analysis fully supported a sister relationship between G. fragrantissima and G. hookeriwithin the Leucothoides clade of Gaultheria. This chloroplast genome will serve as a valuable referencefor future taxonomic and phylogenetic research.

*Gaultheria* Kalm ex L., a genus classified in the family Ericaceae, contains about 288 species (Kron et al. 2020). In Asia, the genus is known for the medicinal species *Gaultheria fragrantissima* Wall., which contains methyl salicylate (oil of wintergreen). This phenolic compound has pharmacological activities is antimicrobial, an insecticide and antioxidant (Mukhopadhyay et al. 2016; Park et al. 2016; Lu et al. 2019). As a Chinese ethnomedicinal plant species, it has been used to treat rheumatism and arthritis due to containing lignan glycoside, flavonoid, organic acid, terpenoid, and steroid, etc. (Ma et al. 2002; Cheng et al. 2009). *Gaultheria fragrantissima* was phylogenetically placed within the Leucothoides clade, but its relationship among species has not been clearly resolved because of possible evolutionary reticulation (Lu et al. 2010, 2019). The chloroplast genome has been successfully utilized for reconstructing phylogenetic relationships in plants (Jung et al. 2014; Huang et al. 2020; Lee et al. 2021). In this study, we analyzed the complete chloroplast genome of *G. fragrantissima* to contribute to the molecular systematics and biology of the species.

The specimen was collected from Dawei Mountain in Pingbian, Yunnan, China (22°55′39″N, 103°41′25″E). A specimen was deposited at the herbarium of Kunming Institute of Botany (collection number: Lu001; contact person: Lu Lu, lulukmu@163.com; https://www.cvh.ac.cn/spms/detail.php?id=ea98cf63) under the voucher number 1251725. Genomic DNA was extracted from fresh leaves using the modified CTAB method (Doyle and Doyle 1987). Illumina Solexa platform (Illumina, San Diego, CA; New England Biolabs, Cedex, France) was used to sequence 150 bp pair-end reads of the 500 bp insert-size libraries. *De novo* assembly was performed with GetOrganelle toolkit (Jin et al. 2020). Reference guided connection and annotation were subsequently conducted using Bandage 0.8.1 (Wick et al. 2015) and Geneious 9.1.4 (Biomatters Ltd., Auckland, New Zealand) with *Vaccinium macrocarpon* Aiton. (NC019616) used as the reference. The plastome structure was further verified by PCR and Sanger sequencing.

Raw reads were deposited in the NCBI Sequence Read Archive (SRA: SRX10204411) and the final annotated chloroplast genome sequence was deposited in NCBI GenBank (accession no. MW563322). The genome sequence is 176,196 bp in total length with a typical quadripartite structure, consisting of a large single-copy region with 107,747 bp (LSC), a small single-copy region with 3509 bp (SSC), and two inverted repeat regions with 32,470 bp (IRs), respectively. The GC content in the chloroplast genome is 36.6%. The plastome comprises 110 unique genes, including 76 protein-coding, 30 tRNA, and 4 rRNA genes.

To identify the phylogenetic position of *G. fragrantissima*, 15 published plastomes from both *Vaccinium* and *Gaultheria*, and the newly obtained plastome were aligned using MAFFT (Katoh et al. 2005). A maximum-likelihood phylogenetic tree was reconstructed by RAxML (Stamatakis, 2014) with 1000 rapid bootstrap replicates and GTR + GAMMA + I substitution model. A sister relationship between *G. fragrantissima* and *G. hookeri* C. B. Clarke in the Leucothoides clade of *Gaultheria* was supported by 100% bootstrap value (Figure 1). These two species were both supported within the Leucothoides clade based on separate and combined DNA sequence data from five genic regions (ITS, *mat*K, *rpl*16, *trn*L-*trn*F, and *trn*S-*trn*G) in Lu et al. (2010) and our study further strongly supported their sister relationship. Our results provide fundamental information for further taxonomic and phylogenetic researches on the Leucothoides clade of *Gaultheria*.

**Figure 1.**
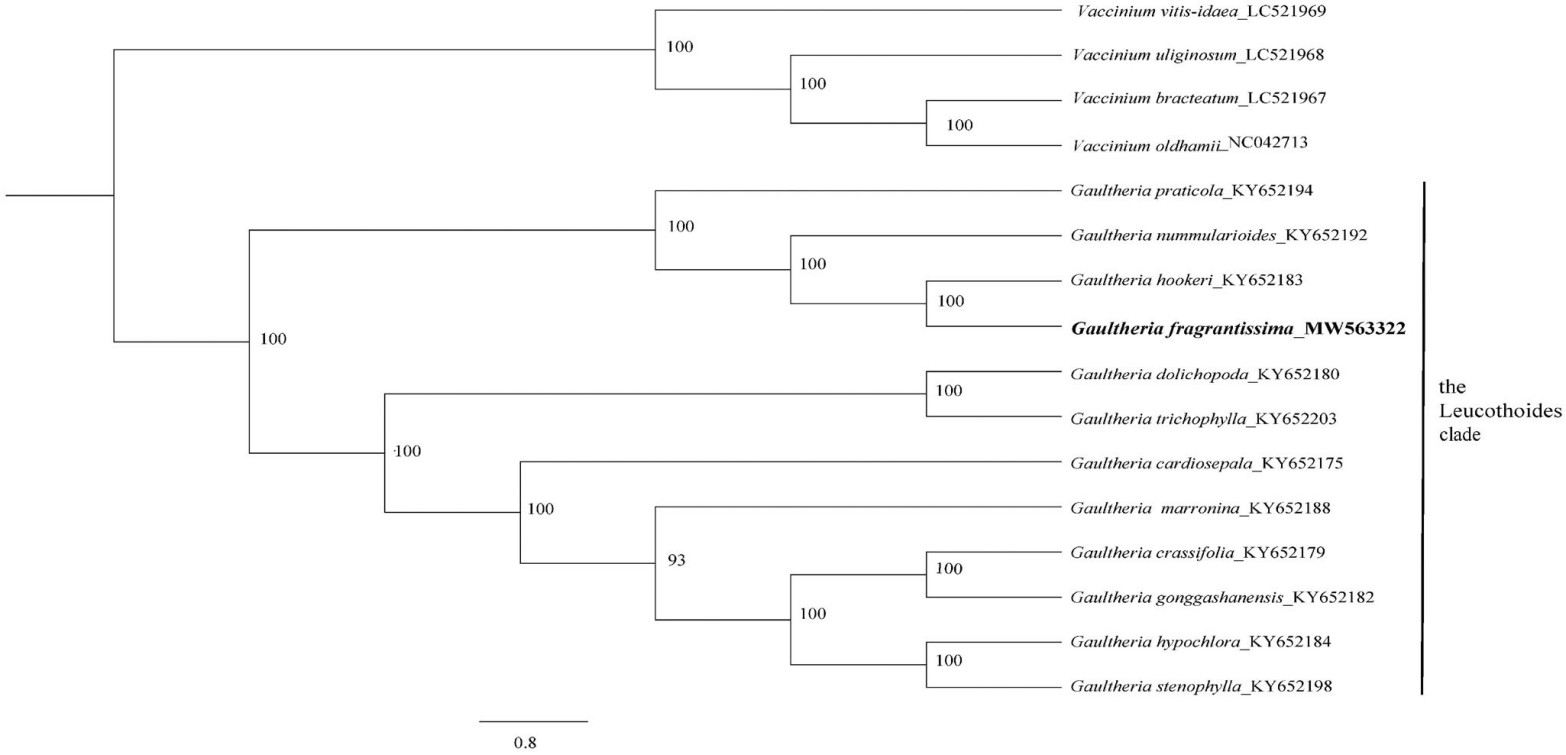
A maximum-likelihood tree based on 16 plastomes reconstructed using RAxML. Bootstrap support values are shown next to the nodes.

## Data Availability

The genome sequence data that support the findings of this study are openly available in GenBank of NCBI at https://www.ncbi.nlm.nih.gov/ under the accession no. MW563322. The associated BioProject, SRA, and Bio-Sample numbers are PRJNA705850, SRX10204411, and SAMN18104638, respectively.
